# Supplementary material for: Predicting visual function by interpreting a neuronal wiring diagram
Source: Nature. 2024 Oct 2;634(8032):113–23. doi: 10.1038/s41586-024-07953-5 (PMC11446822; doi:10.1038/s41586-024-07953-5)
Supplement: Supplementary file 6 — CRF and ERF predictions for individual TmY4 and TmY9 cells. Analogous to Supplementary Data 3, but for TmY target types. Shown are the top four monosynaptic pathways, the strongest pathway passing through each of the top ten intermediary types (ranking from Extended Data Fig. 7), and the trisynaptic pathway Tm1–TmY–Dm3–TmY (see the section entitled Prediction of spatial normalization). [file 41586_2024_7953_MOESM6_ESM.zip › DataS4/TmY9q/720575940642565083.pdf]

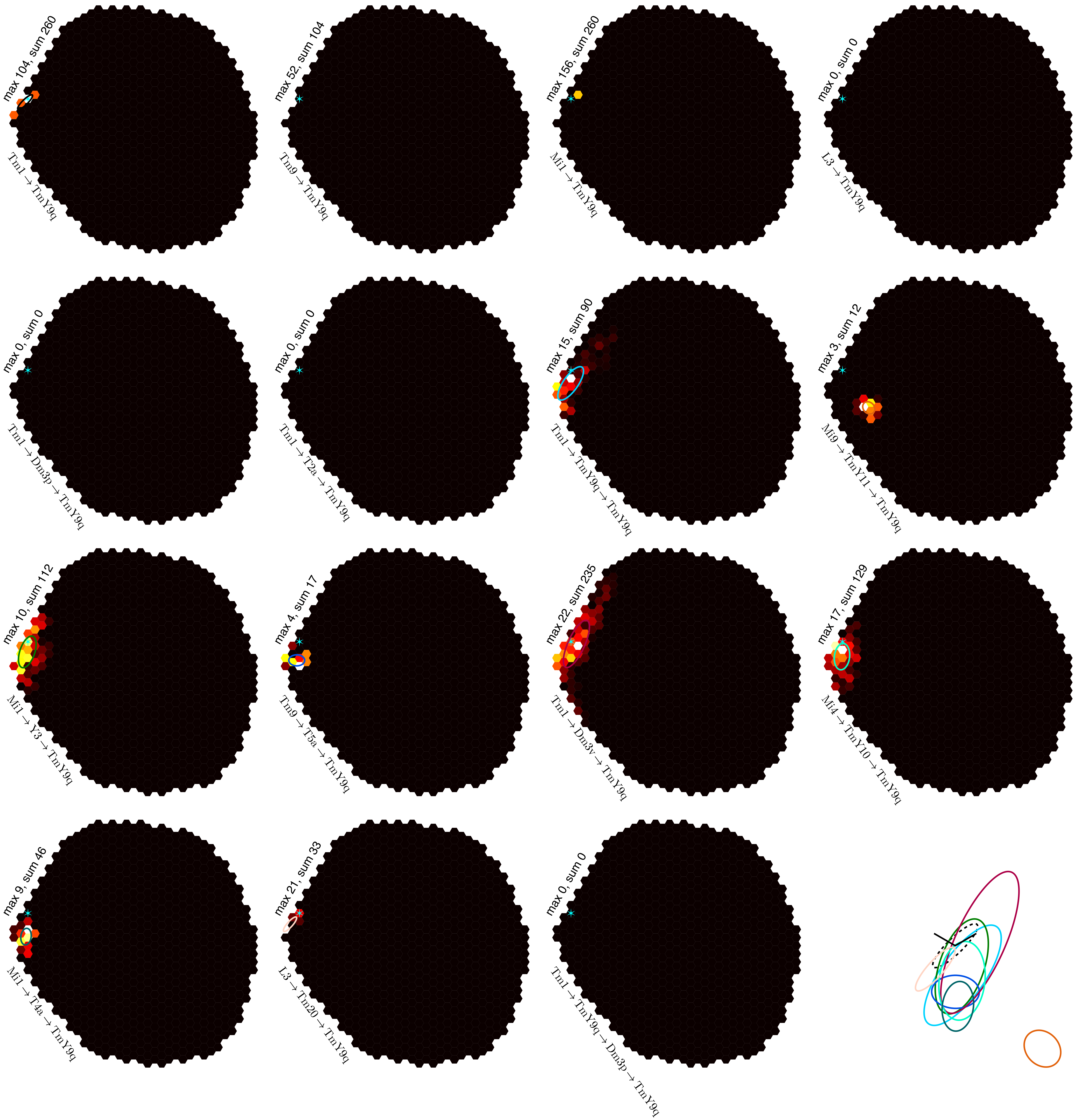
max 104, sum 260
Tm1 → TmY9q
max 52, sum 104
Tm9 → TmY9q
max 156, sum 260
Mi1 → TmY9q
max 0, sum 0
L3 → TmY9q
max 0, sum 0
Tm1 → Dm3p → TmY9q
max 0, sum 0
Tm1 → T2a → TmY9q
max 15, sum 90
Tm1 → TmY9q → TmY9q
max 3, sum 12
Mi9 → TmY11 → TmY9q
max 10, sum 112
Mi1 → Y3 → TmY9q
max 4, sum 17
Tm9 → T5a → TmY9q
max 22, sum 235
Tm1 → Dm3v → TmY9q
max 17, sum 129
Mi4 → TmY10 → TmY9q
max 9, sum 46
Mi1 → T4a → TmY9q
max 21, sum 33
L3 → Tm20 → TmY9q
max 0, sum 0
Tm1 → TmY9q → Dm3p → TmY9q
